# Localization of claudin-2 and claudin-3 in eutopic and ectopic endometrium is highly similar

**DOI:** 10.1007/s00404-020-05472-y

**Published:** 2020-03-05

**Authors:** Alena Hoerscher, Fabian Horné, Raimund Dietze, Eniko Berkes, Frank Oehmke, Hans-Rudolf Tinneberg, Ivo Meinhold-Heerlein, Lutz Konrad

**Affiliations:** 1 Department of Gynecology and Obstetrics, Justus Liebig University, University of Giessen, Feulgenstr. 10-12, 35392 Giessen, Germany; 2 Department of Gynecology, UKE Hamburg, Hamburg, Germany; 3 Gynecological Hospital, Frankfurt, Germany

**Keywords:** Endometrium, Endometriosis, Claudin-2, Claudin-3

## Abstract

**Purpose:**

Claudins as the major components of tight junctions are important in maintaining cell–cell integrity and thus function as a barrier. Dysregulation of the claudins is often associated with loss of the epithelial phenotype, a process called epithelial–mesenchymal transition (EMT), which most often results in gain of migrative and invasive properties. However, the role of claudins in the endometrium or endometriosis has only rarely been examined.

**Methods:**

In this study, we investigated localization of claudin-2 and claudin-3 in the eutopic and ectopic endometrium with immunohistochemistry. A detailed quantification with HSCORE was performed for claudin-2 and claudin-3 in endometrium without endometriosis and in cases with endometriosis compared to the three endometriotic entities: peritoneal, ovarian, and deep-infiltrating endometriosis.

**Results:**

We found a preferential localization of both claudins in the glandular and the luminal epithelial cells in the endometrium with and without endometriosis. Quantification of localization of both claudins showed no differences in eutopic endometrium of control cases compared to cases with endometriosis. Furthermore, both claudins are localized highly similar in the ectopic compared to the eutopic endometrium, which is in clear contrast to previously published data for claudin-3.

**Conclusion:**

From our results, we conclude that localization of claudin-2 and claudin-3 is highly stable in eutopic and ectopic endometrium without any loss of the epithelial phenotype and thus do not contribute to the pathogenesis of endometriosis.

## Introduction

Endometriosis is characterized by the presence of endometrial glands and stroma outside the normal localization, however, irrespective of location, the histological appearance of endometriotic glands always resembles uterine endometrial glands [1]. Despite the histological similarities, it has been suggested that peritoneal endometriosis, endometriomas and deep-infiltrating endometriosis (DIE) are three distinct entities, which do not share a common pathogenesis [2]. Retrograde menstruation followed by implantation of the endometrial tissue on distinct surfaces most often in the pelvic or abdominal cavity is generally accepted as the main cause of endometriosis [3]. Despite the high rate of retrograde menstruation ranging from 76 to 90% [4, 5], only approximately 0.8–2.0% of women in their reproductive age acquire endometriosis as shown in large population-based studies of low-risk patients [6–10]. This discrepancy suggests secondary factors like immune dysfunction for the establishment of endometriotic lesions [11].

Claudins are the major components of tight junctions (TJ), and as transmembrane proteins are mostly located in the apicolateral membranes of epithelial and endothelial cells [12]. The critical contributions of claudins to TJs are to strand formation and the fence and barrier function, although many other proteins are also involved in the structure of TJ complexes. The composition of claudins determines the properties of epithelial barriers such as sealing claudins (claudin-1, -3, -5, -11, -14, and -18) predominating in tight epithelia [13].

In the human endometrium expression of claudin-1, -2, -3, -4, -5, -7, and 10 was found in contrast to claudin-6, -8, -9, -11, -14, and 16–18 [14–18]. In endometriotic lesions, claudin-3 seems to be downregulated [15, 16] similar to claudin-7 [15], whereas claudin-5 mRNA was decreased but the protein levels increased compared to eutopic endometrium [15]. However, as recently published by us, claudin-7 is not downregulated in endometriosis in contrast to subtle changes in localization of claudin-11 [18]. Of note, overexpression of claudin-3 markedly inhibited migration, invasion and epithelial–mesenchymal transition (EMT) of lung squamous cell carcinoma [19].

Similarly, mRNA expression and localization of claudin-4 in eutopic and ectopic endometrium were described controversially. Whereas some researchers found downregulated mRNA and protein expression of claudin-4 in ectopic endometrium [16], others demonstrated no differences [15] or even an increased mRNA expression in ectopic lesions [20]. In endometrial cancer, downregulated claudin-7 has been described to be associated with increased proliferation and metastasis [21].

During the menstrual cycle, an increased abundance of claudin-1 and -5 protein was described for the secretory phase [14]. In contrast, claudin-2, -3 and -4 were similarly abundant throughout the menstrual cycle [14, 16, 17, 22] with the exception that claudin-4 mRNA was significantly higher expressed in the secretory phase compared to the proliferative phase [14]. Intriguingly, claudin-3 protein was expressed in the endothelial cells of the decidual vessels with a possible role in decidual angiogenesis [17]. Progesterone but not estradiol induced claudin-1, -3, -4, and -7 protein expression in human endometrial epithelial cells resulted in downregulation of the barrier, but not fence function [23].

The loss of epithelial cell-to-cell contacts is considered to be one of the hallmarks of EMT, which was suggested to be involved also in the pathogenesis of endometriosis [24]. Recently, we showed no decrease in claudin-7 and only subtle changes in the localization of claudin-11 in ectopic endometrium and suggested that only a partial EMT might be involved in the pathogenesis of endometriosis [18]. Thus, in this study, we examined claudin-2 and claudin-3 expression in eutopic and ectopic endometrium to further clarify the role of cell-to-cell contacts in endometriosis.

## Materials and methods

### Patients

This study has been approved by the Ethics Committee of the Medical Faculty of the Justus-Liebig-University, Giessen, Germany (registry number 95/09). The participants gave written informed consent. All specimens (Table 1) were obtained by hysterectomy (uteri, *n* = 51 patients) or laparoscopy (endometriotic tissues, *n* = 55 with *n* = 59 lesions) from patients mainly suffering from pain. The intraoperative findings were classified according to the revised American Society for Reproductive Medicine score (rASRM) and ENZIAN score [25]. Dating of the endometrial tissue was based on the dates of the last menstrual period and histological evaluation by the pathologist. Although the pathogenesis and definition of DIE is still highly unclear [26], we rely for classification on MRI images and the ENZIAN score [25] which classifies DIE during operation.

**Table 1:** Overview of the tissue samples used for claudin-3

Tissues	Endometrium	Ovarian endometriosis	Peritoneal endometriosis		DIE
All samples	*n* = 51	*n* = 19 (20)	*n* = 17 (18)		*n* = 19 (21)
Median age ± SD	43 ± 7.1	33 ± 4.0	33 ± 4.2		32 ± 5.2
Proliferative (median age)	*n* = 23 (44 ± 8.5)				
Secretory (median age)	*n* = 28 (42 ± 5.9)				
Leiomyoma	*n* = 28				
Adenomyosis	*n* = 10				
Bladder			2		1
Uterosacral lig			1		3
Ovarian fossa			3		
Pouch of Douglas			3		
Round lig of uterus			1		
Peritoneum			1		
Pelvic wall			2		
Rectum					7
Rectosigmoid					2
Rectovaginal septum			1		4
Paraurethral			1		1
Sigmoid colon			1		1
Vagina					1
Intestine					1
Mesovarium			1		
Lig latum uteri			1		

e.g. *n* = 19 (20) means 20 lesions from 19 patients

*lig*, ligament; *DIE*, deep-infiltrating endometriosis

Specimens were fixed in Bouin’s solution (and partly in formaldehyde for the histological evaluation by the pathologist) and embedded in paraffin. After staining 5 µm sections with hematoxylin and eosin, the histological evaluation was performed.

### Immunohistochemical analysis and quantification

Serial sections of 5 µm were cut to ensure that in most cases, the same lesions could be examined. Immunohistochemistry (IHC) of bouin-fixed or formalin-fixed specimens was performed as published previously [27]. The EnVision Plus System (cat-no K4002, DAKO, Hamburg, Germany) was used according to the manufacturer’s instructions. Briefly, antigen retrieval was performed with a citrate buffer (pH 6, DAKO) and then the jars containing the slides were put into a steamer (Braun, Multi Gourmet) at 100 °C for 20 min and remained in the steamer for cooling for 20 min. Primary antibodies against claudin-2 (diluted 1:200, cat-no 32-5600, Thermo Fisher, Waltham, MA, USA) or claudin-3 (diluted 1:100, cat-no 34-1700, Invitrogen, Waltham, MA, USA) were added and incubation was done in a humidified chamber overnight at 4 °C. After washing with PBS, incubation with the appropriate secondary antibody (cat-no K4002, DAKO) was done for 30 min at room temperature. The staining was visualized with diaminobenzidine (Liquid DAB K3467, DAKO). Counterstaining was performed with Mayer’s hematoxylin (Waldeck, Germany) and after dehydration in ethanol, slides were mounted with Eukitt. Negative controls for IHC were prepared by omission of the primary antibody. Digital images were obtained with Leica DM 2000/Leica MC170/Leica application suite LAS 4.9.0 and then processed with Adobe Photoshop CS6. IHC quantification was done by use of the HSCORE (0, no staining; 1 + , weak, but detectable; 2 + , moderate or distinct; 3 + , intense) which was calculated for each tissue by summing the percentages of cells grouped in one intensity category and multiplying this number with the intensity of the staining. All glands or cysts were used for evaluation of the HSCORE.

### Statistics

All values are presented as mean ± standard error of the mean (SEM) or median. HSCORE values between the different groups were analysed using one-way analysis of variance (ANOVA). Then, comparison between more than two groups was done with the test of Kruskal–Wallis. *P* values ≤ 0.05 were considered to be significant. GraphPad Prism 6.01 (www.graphpad.com) was used for the statistics.

## Results

**Fig. 1:**
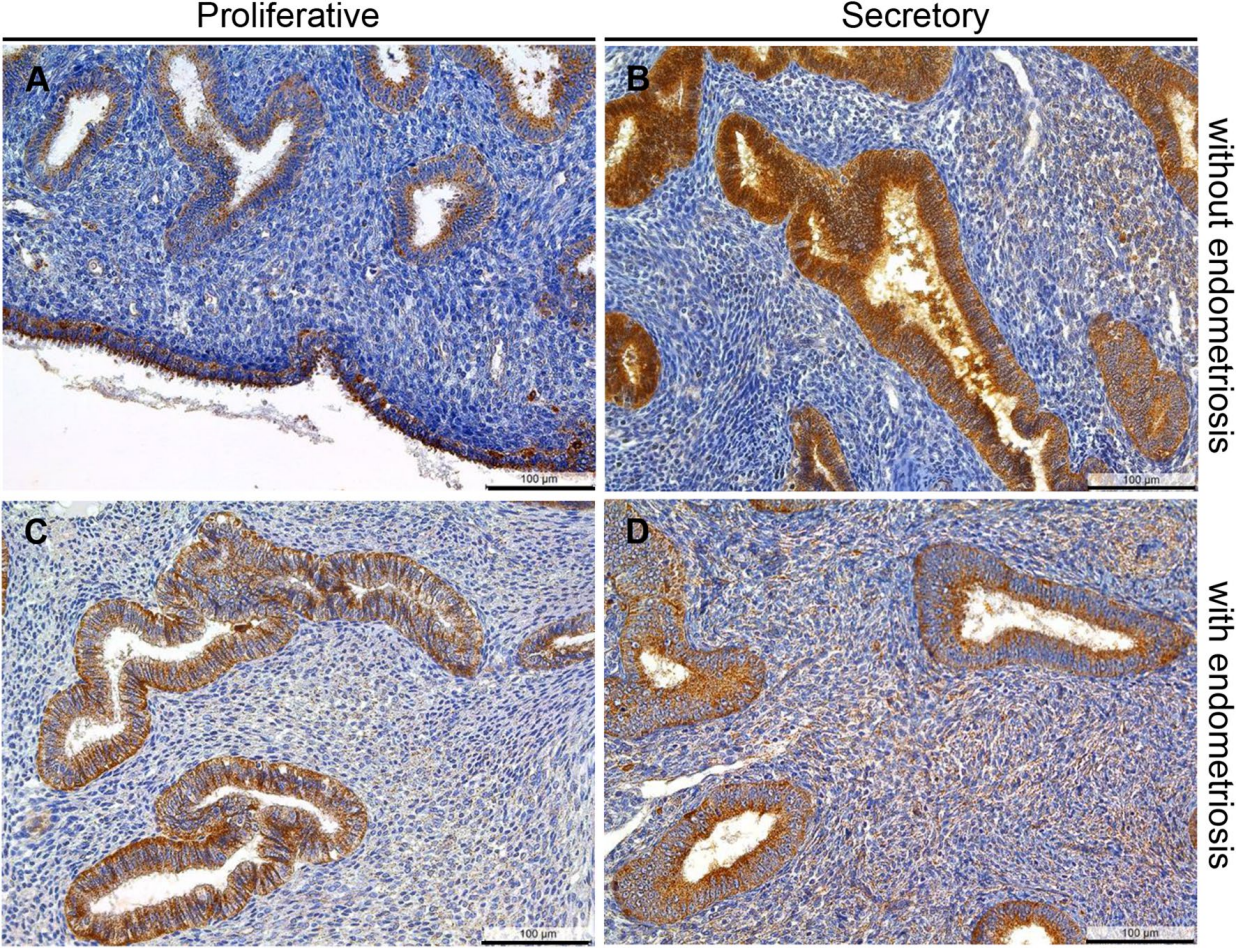
Representative microphotographs of claudin-2 in proliferative (**a**) and secretory (**b**) endometrium without endometriosis and in proliferative (**c**) and secretory (**d**) endometrium with endometriosis. Similarly, the luminal cells are also stained (**a**). Most samples showed a preferential apical staining. An example of a negative control can be found in Fig. 3f. Scale bars 100 µm, magnification × 200

Analysis of claudin-2 in patients with and without endometriosis showed a preferential apical localization for glandular and luminal eutopic epithelial cells in the majority of epithelial cells and glands in both the proliferative and the secretory phase (Fig. 1a−d). We found a high similarity of the HSCORE between patients with and without endometriosis as well as proliferative and secretory phases (Table 2).

**Table 2:** Comparison of localization of claudin-2 and claudin-3 in endometrium with and without endometriosis by the HSCORE

	Endometrium without endometriosis		Endometrium with endometriosis	
	Proliferative	Secretory	Proliferative	Secretory
Claudin-2				
*N *(median age)	6 (45.5)	6 (46.5)	6 (32.5)	8 (42)
Mean	165	187	193	185
SEM	22.2	24.2	9.5	6.0
*P*		ns	ns	ns
Claudin-3				
*N* (median age)	9 (46)	10 (40.5)	14 (41.5)	18 (42.5)
Mean	268	253	275	265
SEM	11.2	12.0	8.5	9.4
*P*		ns	ns	ns

*ns*, not significant

**Fig. 2:**
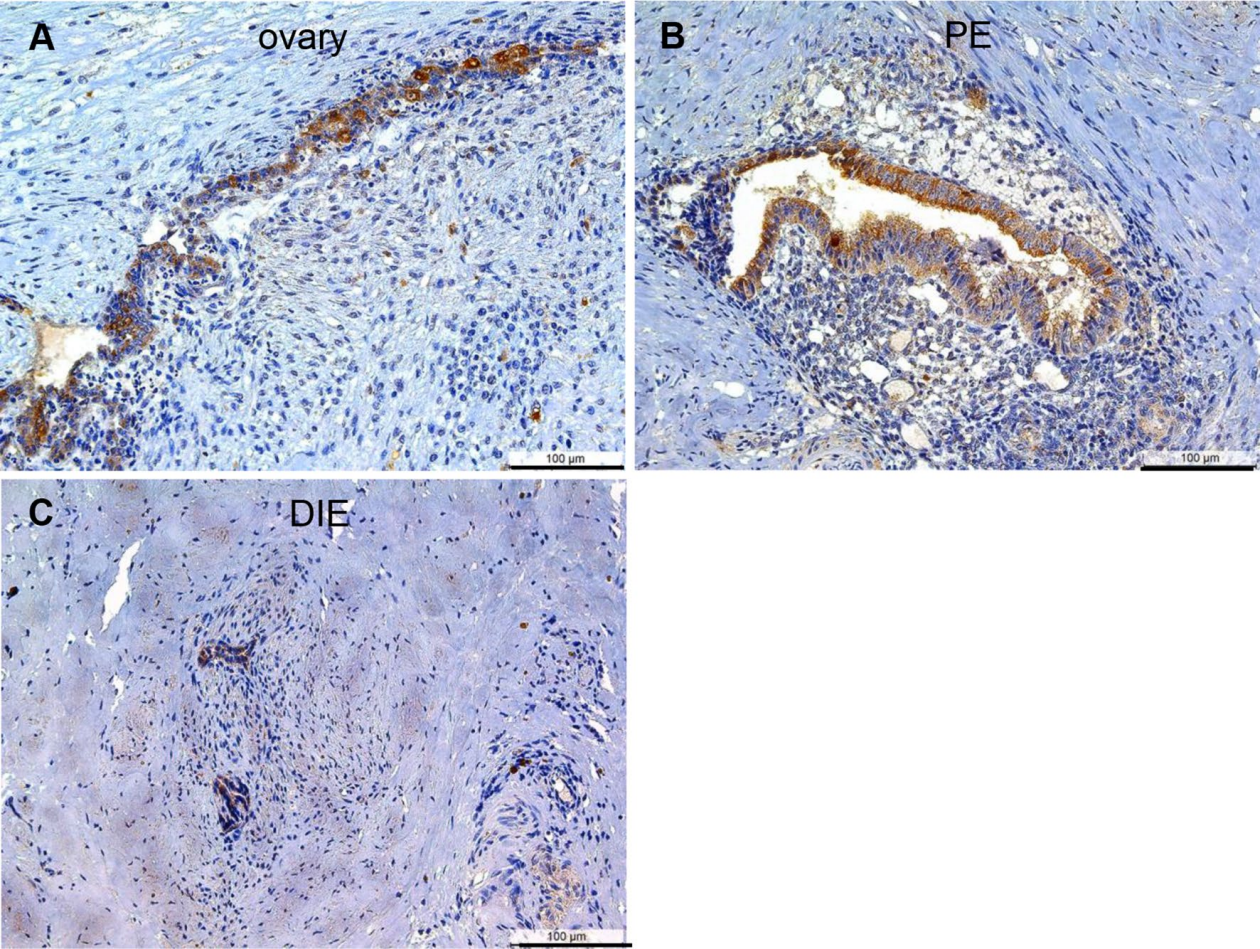
Representative microphotographs of claudin-2 in ovarian endometriosis (**a**), peritoneal endometriosis (**b**, bladder), and DIE (**c**, rectovaginal septum). In Fig. 2b, the preferential apical staining is best visible. PE, peritoneal endometriosis; DIE, deep-infiltrating endometriosis. Scale bars 100 µm, magnification × 200

Positivity for claudin-2 was further identified in the majority of endometriotic epithelial cells and lesions irrespective of the three endometriotic entities: ovarian (Fig. 2a), peritoneal (Fig. 2b) or deep-infiltrating endometriosis (Fig. 2c). As the HSCORE showed no significant differences between eutopic endometrium with and without endometriosis (Table 2), we merged both datasets and found no significant differences between the eutopic and ectopic endometrium (Table 3).

**Table 3:** Comparison of localization of claudin-2 and claudin-3 in eutopic and ectopic endometrium by the HSCORE

	Endometrium	Ovarian EM	DIE	Peritoneal EM
Claudin-2				
*N* (median age)	26 (42)	6 (33)	6 (35.5)	7 (33)
Mean	183	179	209	220
SEM	7.8	25.1	25.2	31.2
*P*		ns	ns	ns
Claudin-3				
*N* (median age)	51 (43)	20 (33)	25 (32)	17 (32)
Mean	266	266	286	263
SEM	5.0	8.6	4.6	9.5
*P*		ns	ns	ns

*N* = number of lesions; *EM*, endometriosis; *ns*, not significant

**Fig. 3:**
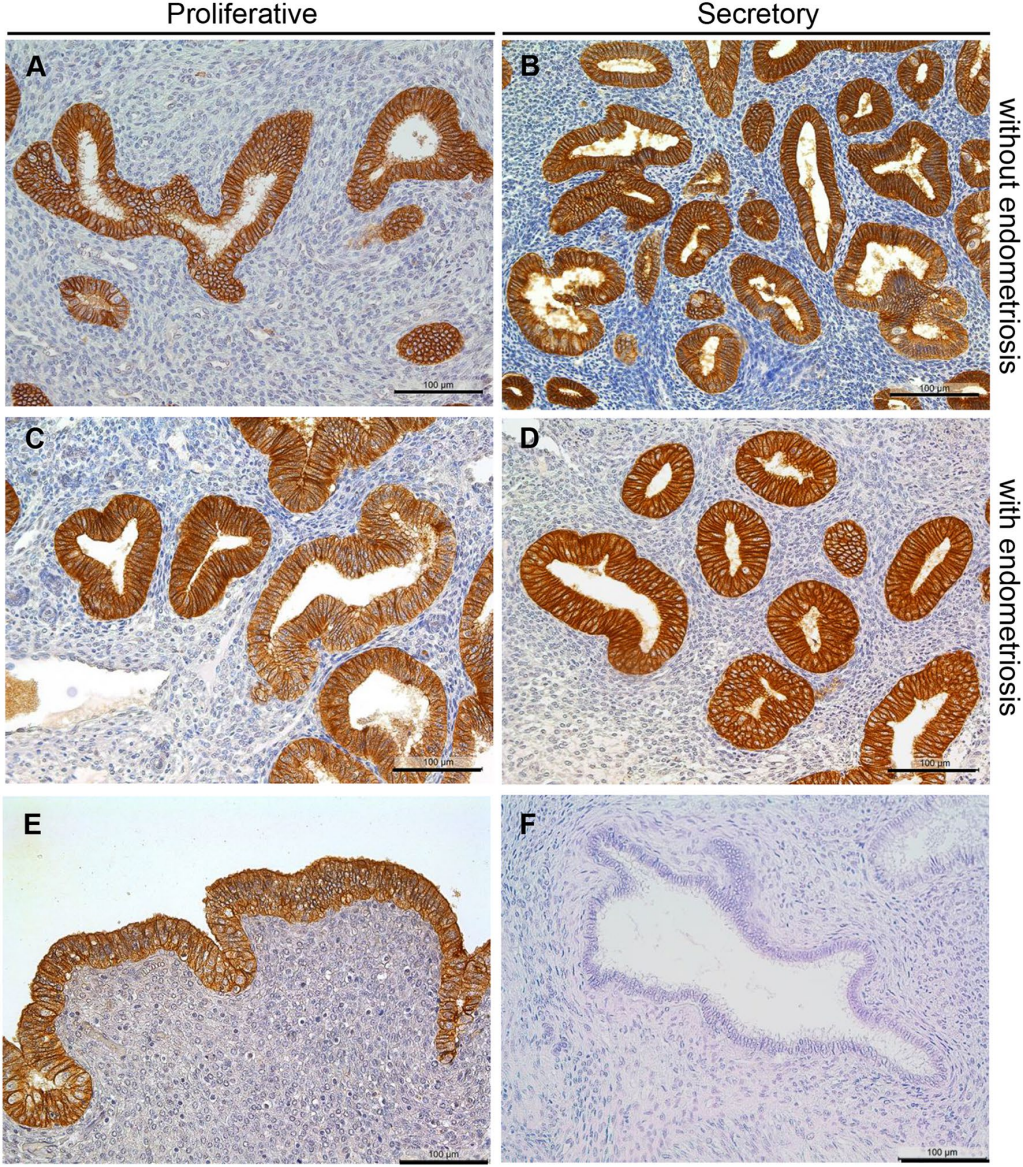
Representative microphotographs of claudin-3 in proliferative (**a**) and secretory (**b**) endometrium without endometriosis and in proliferative (**c**) and secretory (**d**) endometrium with endometriosis. In all samples, a strong membranous staining is visible. Similarly, the luminal cells are also stained (**e**); the negative control showed no staining (**f**). Scale bars 100 µm, magnification × 200

Localization of claudin-3 in patients with and without endometriosis was strong in the membranes of glandular eutopic epithelial cells, nearly approaching 100% of all epithelial cells in both the proliferative and the secretory phase (Fig. 3a–d). A strong membrane localization was also found in the luminal cells (Fig. 3e), whereas the negative control of the same patient showed no staining (Fig. 3f). Quantification of the staining showed a high similarity of the HSCORE between patients with and without endometriosis as well as proliferative and secretory phases (Table 2).

**Fig. 4:**
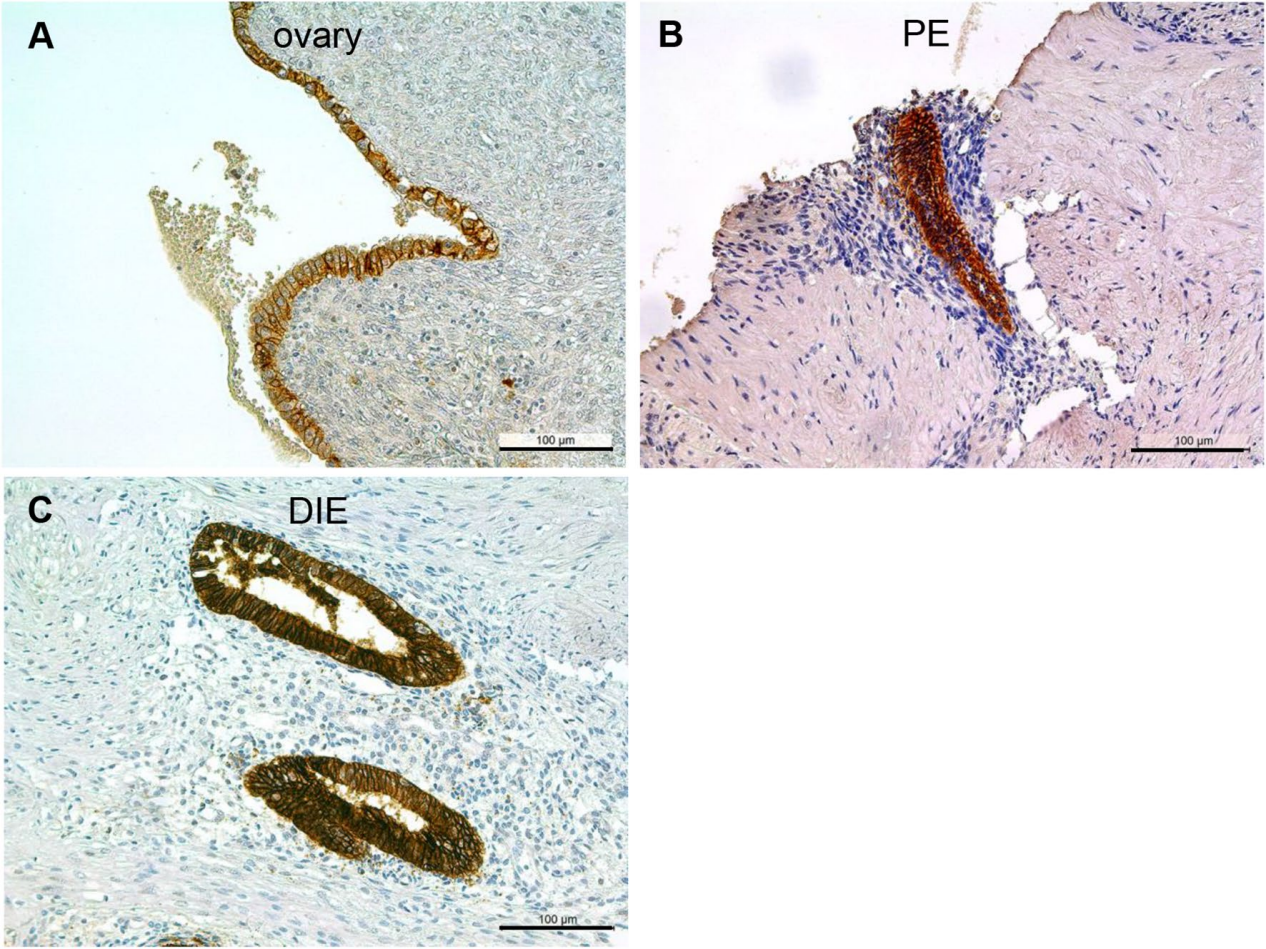
Representative microphotographs of claudin-3 in ovarian endometriosis (**a**), peritoneal endometriosis (**b**, Douglas), and DIE (**c**, rectum). In all samples, a strong membranous staining is visible. PE, peritoneal endometriosis; DIE, deep-infiltrating endometriosis. Scale bars 100 µm, magnification × 200

Positivity for claudin-3 was further identified in almost all ectopic endometriotic epithelial cells and lesions irrespective of the three endometriotic entities: ovarian (Fig. 4a), peritoneal (Fig. 4b) or deep-infiltrating endometriosis (Fig. 4c). As the HSCORE showed no significant differences between eutopic endometrium with and without endometriosis (Table 2), we merged both datasets and found no significant differences between the eutopic and ectopic endometrium (Table 3).

## Discussion

In this study, we analyzed epithelial cells of eutopic endometrial glands and endometriotic lesions in the ovary, peritoneum, and DIE with claudin-2 and claudin-3. Our results demonstrate convincingly that both claudins are ubiquitously expressed in both the eutopic as well as the endometriotic lesions with a highly similar pattern and abundance.

Claudin-2 is expressed in epithelial layers with high paracellular permeability such as proximal tubules and the intestine [28]. Mostly, an overexpression of claudin-2 in gynecological tumors has been observed [29]. In the human endometrium, claudin-2 is expressed similarly in the proliferative and secretory phase [14] as described in our study. Furthermore, we identified a stable expression of claudin-2 also in the ectopic endometrium compared to the eutopic endometrium which is according to our knowledge, a new finding. The observed preferential localization in the apical region is similar to the apical localization in intestinal cell organoids [30].

In the human endometrium with endometriosis, an impaired expression of claudin-3, -5 and -7 has previously been identified [15]. In another study, claudin-3 and claudin-4 have been described to be downregulated in ectopic endometrium [16]. Both suggested that the downregulation of various members of the claudin family might contribute to endometrial cell detachment and increase the number of cells invading pelvic organs [15, 16]. These data are in clear contrast to our results which did not show any dysregulation of claudin-3 in endometriotic lesions. However, in our study, we demonstrated localization of claudin-3 in the endometrial luminal epithelial cells which is in accordance to observations by Schumann et al. [17], whereas in the other studies, it was not mentioned [14–16]. Furthermore, we recently could not confirm downregulation of claudin-7 in endometriotic lesions as published by Gaetje et al. [15], but found a moderate non-significant increased presence in peritoneal and DIE lesions instead [18]. Although we supposed that this might be due to different detection systems and antibodies used, we found that tissue fixation with Bouin was superior to formalin in some cases of claudin detection (unpublished observation).

In numerous types of human cancer, the number of cell–cell junctions decreases, permitting the escape of cancer cells from their primary sites, along with the acquisition of invasive and metastatic properties [31, 32]. However, in endometrioid endometrial cancer, an upregulation of claudin-2 [29] as well as of claudin-3 [29, 33–35] was described. Thus, it remains unclear why in endometriosis, a downregulation of claudin-3 [15, 16] should contribute to the dissemination of endometrial cells.

EMT is a biological process characterized by two hallmarks, loss of the epithelial phenotype and gain of properties of mesenchymal cells [31, 32, 36]. This process requires a series of complex changes in cell architecture and behavior which is driven by various cellular signals. The molecular changes of this transition include the loss of epithelial markers such as E-cadherin, keratins, desmoplakin, mucin-1, occludin and claudins and gain of mesenchymal markers like N-cadherin, α-smooth muscle actin, vimentin and fibronectin. Alterations of these pathways are associated with enhanced migration, invasiveness and resistance to apoptosis [31, 32, 36].

Recently, EMT was described to be involved in endometriosis [24, 37]. Although endometriotic cells share some similarities with metastatic tumor cells in terms of dissemination and invasion [24], endometrial cells do not necessarily need to undergo EMT, as menstruation allows a physiological detachment of cells. Additionally, irrespective of the location of endometriotic lesions such as in the ovary, peritoneum, or DIE, ectopic endometriotic glands nearly always have an overtly endometrioid appearance and histologically resemble uterine endometrial glands, clearly indicating no loss of the epithelial phenotype [1]. Recently, we observed no loss of the endometrial epithelial phenotype as defined by expression of keratins and claudins in endometriosis and suggested that if at all only a partial EMT might be involved in the pathogenesis of endometriosis [18, 27]. The current study further corroborates this assumption by showing a stable expression of claudin-2 and claudin-3 in eutopic and ectopic endometrium.

Although many authors stress the differences of eutopic and ectopic endometrial cells, however, we and others [38] believe that these differences may be explained as a direct consequence of the different environments, such as the peritoneal fluid and the intraovarian microenvironment of the lesions, in relation to the intrauterine environment. We suggest that the changes in the eutopic endometrium at the beginning of the disease are quite subtle and that the majority of differences can be observed after the implantation. Based on our observations about the similarities between eutopic and ectopic endometrial epithelial and stromal cells [27, 39], we propose to focus on the interplay of endometrial cells with, for example, peritoneal cells at the site of implantation for future research on the pathogenesis of endometriosis [40].

In summary, in our study, we could clearly show no loss of cell-to-cell contacts characterized by a stable localization of claudin-2 and claudin-3 in epithelial cells of both eutopic and ectopic endometrium. Thus, the epithelial phenotype is definitely not lost and only a partial EMT might contribute to the pathogenesis of endometriosis.
